# Investigation on Green Synthesis, Biocompatibility, and Antibacterial Activity of Silver Nanoparticles Prepared Using *Cistus incanus*

**DOI:** 10.3390/ma14175028

**Published:** 2021-09-02

**Authors:** Wioletta Florkiewicz, Klaudia Pluta, Dagmara Malina, Karolina Rudnicka, Anna Żywicka, Martin Duarte Guigou, Bożena Tyliszczak, Agnieszka Sobczak-Kupiec

**Affiliations:** 1 Department of Materials Science, Faculty of Materials Engineering and Physics, Cracow University of Technology, 37 Jana Pawła II Av., 31-864 Krakow, Poland; bozena.tyliszczak@pk.edu.pl (B.T.); agnieszka.sobczak-kupiec@pk.edu.pl (A.S.-K.); 2 Department of Chemical Technology and Environmental Analytics, Cracow University of Technology, 24 Warszawska St., 31-155 Krakow, Poland; klaudia.pluta@pk.edu.pl (K.P.); dagmara.malina@pk.edu.pl (D.M.); 3 Department of Immunology and Infectious Biology, Faculty of Biology and Environmental Protection, University of Lodz, 12/16 Banacha St., 90-237 Lodz, Poland; karolina.rudnicka@biol.uni.lodz.pl; 4 Department of Microbiology and Biotechnology, Faculty of Biotechnology and Animal Husbandry, West Pomeranian University of Technology, Szczecin, Piastów 45, 70-311 Szczecin, Poland; anna.zywicka@zut.edu.pl; 5 Facultad de Ingeniería y Tecnologías, Universidad Católica del Uruguay, B de Octubre 2738, Montevideo CP 11600, Uruguay; martin.duarte@ucu.edu.uy

**Keywords:** silver nanoparticles, green synthesis, biocompatibility, antibacterial efficacy

## Abstract

This paper describes the plant-mediated preparation of silver nanoparticles with aqueous extract and infusion of *Cistus incanus* leaves. To evaluate aqueous extract and infusion antioxidant capacity and total phenolic content the DPPH and Folin–Ciocalteau methods were utilized. The antioxidant capacity and total phenolic content of extract and infusion were equal to 85.97 ± 6.54 mg gallic acid equivalents per gram of dry weight.; 10.76 ± 0.59 mg/mL and 12.65 ± 1.04 mg gallic acid equivalents per gram of dry weight.; 3.10 ± 0.14 mg/mL, respectively. The formed nanoparticles displayed the characteristic absorption band in the 380–450 nm wavelength range. The average size of particles was in the 68.8–71.2 nm range. Morphology and phase composition analysis revealed the formation of spherical nanoparticles with a face-centred cubic structure. Immune compatibility tests of nanoparticles and plant extracts showed no activation of the THP1-XBlue™ monocyte. Cytotoxicity tests performed with L929 mice fibroblasts showed that nanoparticles should be utilized at a concentration of 16 ppm. The minimum inhibitory concentrations determined with the microdilution method for nanoparticles prepared with plant infusion for *S. aureus* and *S. epidermidis* were 2 ppm and 16 ppm, respectively.

## 1. Introduction

The synthesis of nanoscale materials is an emerging area of science and technology and has focused researchers’ attention, owing to nanomaterials’ immense potential applications in various fields of life. Nanosized materials may contribute solutions to technological and environmental demands in the fields of energy [1], water treatment [2] catalysis [3], and biomedical sciences [4,5,6]. One of the most demanding aspects of nanotechnology is the preparation of monodisperse materials of controllable size, shape, and chemical composition. Nanomaterials can be synthesized utilizing a wide range of methods, some, however, require the usage of toxic chemicals and result in the releasing of hazardous by-products. Therefore, there is a fundamental need to develop clean, environmentally benign, non-toxic, and safe approaches to the preparation of nanomaterials exploiting sustainable and cost-effective technologies. These essential challenges have led researchers to place a focus on biological systems, including bacteria [7], fungi [8], algae [9], and plants [10]. Considering the cost-effectiveness of the preparation of nanoparticles (NPs), methods based on phytochemical-mediated reduction of metal ions may provide an economic and valuable alternative for the production of NPs [11]. Numerous plant metabolites, e.g., phenolic compounds [12], terpenoids [13], proteins [14], and co-enzymes [15], play a crucial role in reduction of metal ions and stabilization of NPs. Applying plant metabolites to NPs’ synthesis provides an easy, single-step, inexpensive, and environmentally benign methods for nanometric structure obtaining, eliminating the use of hazardous solvents and the time-consuming culturing of fungi, algae, etc. [16]. It is worth emphasizing that reducing and capping abilities of plant products also decrease the production costs of nanostructures, as there is no need to use additional stabilizing agents [17].

For millennia, plant-derived natural products have played a significant role in medical treatments and disease prevention. The earliest world records date from around 2600 BCE and report the application of plants in medicine coming from Mesopotamia. Interestingly, some of the proposed solutions are still used today in the treatment of a wide range of illnesses including cold, inflammation, and parasitic infection [18]. Moreover, in recent decades, around 71% of pharmaceuticals have been obtained from natural products [19]. Additionally, recent studies show potential anti-cancer and immunomodulatory effects of some plant extracts rich in terpenes, terpenoids, and phenolics [20].

Phytochemical-mediated synthesis with the extracts obtained from a wide spectrum of plant species were particularly focused on silver and gold nanoparticle preparations. *Cistus incanus*, also known as pink or hairy rockrose, *Cistus creticus* or *Cistus villosus* is one of the medicinal plants of the greatest therapeutic and reducing potential [21]. Herbal teas, prepared from aerial parts of *C. incanus* due to well-reported antioxidant [22], anti-inflammatory [23], antiviral [24], and antimicrobial [25] properties, have become popular as a supplement to an everyday diet. Moreover, numerous reports indicate that natural products containing polyphenolic compounds exhibit anticaries activities and can be used for their prophylaxis [26]. Analysis of the polyphenolic composition of an aqueous extract of *C. incanus* shows that its pronounced antioxidant activity is based on three major groups of compounds: tannins, flavonoids, and phenolic acids derivatives. Nine different flavonoids were identified in *C. incanus* extract, including four flavanols and five flavonols derivatives, such as rutin, myricitrin, and quercitrin. On the other hand, the tannin content was negligible—only gallic acid and hexahydroxydiphenoyl-glucose were identified. In the third class of compounds, only uralenneoside was found in this extract [27].

Some studies showed that *C. incanus* is a promising material for the production of NPs. Klekotko et al. [28] used *C. incanus* aqueous extract for preparation of popcorn-shaped gold nanoparticles (AuNPs). The morphology of the obtained AuNPs was characterized under transmission electron microscope and multiphoton-excited luminescence properties were examined. However, a biological assessment of the obtained AuNPs was not performed. Jing et al. [29] reported on biosynthesis of copper oxide NPs with *C. incanus* aqueous leaf extract. The prepared NPs were characterized with scanning electron microscopy, X-ray diffractometry, and UV-Visible spectroscopy. Moreover, potential effect on alloxan-induced oxidative stress conditions during cardiac injury in animals was examined. Nonetheless, to the best of our knowledge and literature survey, *C. incanus* has not been used for the preparation of silver nanoparticles (AgNPs) thus far. We want to emphasize that the combination of the antimicrobial properties of AgNPs and the health-promoting properties of *C. incanus* may create new possibilities for the application of such-obtained nanoparticles, and may also reveal potential multifunctionality in various fields of medicine.

In this study, an attempt was made to develop accessible, cost-efficient, and environmentally friendly biosynthesis of AgNPs using *C. incanus* aqueous extraction and infusion, taking into consideration this plantantioxidant potential. We focused on examining the influence of plant extracts and infusions on the stability of nanoparticles and characterization of radical scavenging potential of *C. incanus*. For this purpose, AgNPs suspensions were prepared using plant extracts and infusions at various concentrations for the sake of optimization of the route of AgNPs obtaining. Finally, the produced NPs as well as plant extracts and infusions were tested for cytotoxicity and immune compatibility, and the antibacterial efficacy of AgNPs was investigated.

## 2. Experiment Methods

### 2.1. Preparation of Plant Extracts and Infusions

*C. incanus* leaves’ extract and infusion were utilized to obtain AgNPs suspensions, given the antioxidant and health-promoting properties of the plant. *C. incanus* dried and ground leaves were purchased from a local health food store. For the purpose of aqueous extract and infusion obtaining, 10 g of finely cut leaves and 150 mL distilled water were used. Leaves extraction was performed using a Soxhlet apparatus for 10 h. For plant-infusion preparation, 10 g of dry leaves of *C. incanus* were immersed in 150 mL of hot distilled water (80 °C ± 3 °C) and extracted for 30 min in a glass beaker. Such prepared plant extracts were stored at 4 °C and used to produce the AgNPs suspensions.

### 2.2. Obtaining Silver Nanoparticles 

AgNPs suspensions were prepared using silver nitrate (POCH S.A.) as a source of Ag ions. In order to conduct the phytochemical-mediated reduction of Ag^+^, 5 mL, 10 mL and 15 mL of plant solutions were added, dropwise to 45 mL, 40 mL and 35 mL of AgNO_3_ solutions, respectively. All solutions consisted of 500 ppm of Ag^+^. The nanoparticles synthesis was performed under constant stirring. A reduction of silver ions to AgNPs was confirmed by solutions’ color changes from colorless to brown. The obtained AgNPs suspensions were kept at 4 °C.

### 2.3. Total Phenolic Content and Antioxidant Capacity of Extracts

In order to examine the radical scavenging ability (RSA) of the obtained plant extracts and infusions 1,1-diphenyl-1-picrylhydrazyl (DPPH) radical was applied. The measurement of the total phenolic content (TPC) was performed applying the Folin–Ciocalteau (F-C) manner. DPPH radical can be reduced by the active compounds extracted from plants, resulting in a change of color of DPPH ethanol (96%, POCH S.A.) solution from purple to yellow, or even solution discoloration. The reduction of this radical leads to decreased maximum absorption, and this change, which is proportional to the amount of the reduced radical, can be measured with a spectrophotometer [30]. In order to prepare a radical solution, 19.71 mg of DPPH radical (Sigma-Aldrich, St. Louis, MO, USA) was dissolved in 100 mL of ethanol. Subsequently, an appropriate amount of ethanol was added to such prepared DPPH solution to reach the absorbance signal of approximately 0.9 at 517 nm. First, the absorbance of the control sample (A_0_) obtained by mixing 1.5 mL of the prepared DPPH solution and 20 μL of ethanol (96%) was measured. The tested specimens were prepared by adding 20 μL of various concentrations (2 mg/mL d.w. to 12 mg/mL d.w.) of the extracts to 1.5 mL of DPPH solution. After 15 min of incubation at room temperature, the absorbance was measured against ethanol, as a blank, at 517 nm. Antioxidant activity was expressed as the extract concentration necessary to neutralize 50% of free DPPH radicals (IC_50_). The IC_50_ was calculated by plotting the correlation between the concentration of the extract (mg/mL) and inhibitory concentration (IC, %). The measurements were conducted in triplicate and the average value (A) was determined. The results were calculated according to Equation (1).

(1) $$\mathrm{IC}\;\left[\%\right]=\frac{A_{0}-A}{A_{0}}\times 100$$

The total phenolic content (TPC) in extract and infusion was tested with the colorimetric F-C assay. This method relies on transfer of electrons from phenolic compounds to F-C reagent resulting in blue color formation. The color intensity is proportional to phenolic concentration, thus phenolic content can be analyzed by measurement of absorbance. As a standard in spectrophotometric measurement gallic acid (Acros Organics) was applied. The TPC analysis’ results were presented as gallic acid equivalents (GAE, mg·g^−1^). Calibration standards were prepared in a concentration range of 0.05; 0.15; 0.25; 0.35; 0.5 mg·mL^−1^ using gallic acid aqueous solution (5 mg·mL^−1^) as a working solution. Calibration solutions were prepared in a cuvette by mixing calibration standards (20 µL), double distilled water (1.58 mL), and F–C reagent (100 µL). After 3 min 300 µL of saturated solution of Na_2_CO_3_ (POCH S.A.) was added to the cuvette. The prepared solutions were thermostatically maintained at 40 °C for 30 min. The analyzed specimens were obtained in an identical procedure using 20 µL of the tested sample instead of a standard solution. The measurements of absorbance were accomplished in triplicates at 765 nm against a blank sample containing no gallic acid.

### 2.4. Size, Shape and Phase Composition of AgNPs

To confirm AgNPs formation in accordance with the proposed method and assessment of stability of the prepared suspensions UV–Vis spectral analysis was utilized. The measurement was carried out in a wavelength range of 300 nm to 750 nm with an Evolution 220 spectrophotometer. All measurements were performed at room temperature. The average particle size and the size distribution of obtained NPs were determined with the dynamic light scattering (DLS) method, using a Zetasizer Nano ZS apparatus (Malvern Panalytical, Malvern, United Kingdom). Analysis was performed at room temperature. The phase composition analysis of AgNPs was carried out employing XRD-Philips X’Pert diffractometer (Phillips, Almelo, Netherlands) with Cu Kα radiation in 2θ range of 10° to 90°. Imaging and analysis of the morphological features of the AgNPs were taken with SEM Zeiss Ultra Plus microscope (Carl Zeiss, Oberkochen, Germany) combined with an EDS microanalysis system Quantax 400 V (Billerica, MA, USA ). To perform SEM-EDS analysis, AgNPs sample was placed on an Si wafer.

### 2.5. FT-IR Analysis

The functional groups and composition of the extracts and infusions were characterized by Fourier transform infrared spectroscopy, in the range of 4000 cm^−1^ to 400 cm^−1^ utilizing a Thermo Scientific Nicolet iS5 FTIR spectrometer (Thermo Fisher Scientific, Waltham, MA, USA) equipped with iD7 ATR accessory. FTIR spectral measurements were performed in the interest of identifying potential biomolecules in the extracts and infusions responsible for Ag^+^ reduction and the capping of AgNPs.

### 2.6. Cytotoxicity Test

Considering prospective biomedical applications, it is necessary to access toxicity of extracts and NPs at the in vitro level, which is essential to establishing safe doses of substances that can be used for further research. Selected infusion and extract of *C. incanus* leaf, as well as AgNPs suspensions, were subjected to cytotoxicity tests, according to the international protocol for the cytotoxic effect assessment of devices dedicated to medical application (ISO 10993-2009-5). The cytotoxicity was evaluated after 24 h incubation of NCTC clone 929: CCL 1 (L929 fibroblasts) with an aqueous *C. incanus* extract and infusion and their nanoparticle suspensions in MTT (3-(4,5-dimethylthiazol-2-yl)-2,5-diphenyltetrazolium bromide) colorimetric assay. Serial two-fold dilutions were prepared from the *C. incanus* extract and infusion and NP suspensions to obtain concentrations of 0.003% to 100% for *C. incanus* solutions and 0.015 ppm to 500 ppm for AgNPs. Two technical experiments were performed, each repeated four time per sample.

### 2.7. Immunocompatibility Assay

To examine the immunostimulatory effects, THP1-XBlue™ human monocytes were used (Invitrogen, San Diego, CA, USA). This recombinant cell line is constructed to detect any immunostimulant that via toll-like receptors (TLR), and activates the κ-light-chain-enhancer of activated B (NF-κb). The quantification of NF-κB induction was performed in accordance with Florkiewicz et al. [31]. All prepared samples were tested in dilutions selected based on cytotoxicity studies results.

### 2.8. Antimicrobial Activity of AgNPs

The in vitro activity of AgNPs against bacterial strains was evaluated in compliance with the international standard protocol ISO 20776-1. The broth microdilution procedures described in this part of the ISO standard were used to determine the minimum inhibitory concentrations (MICs), defined as the lowest concentration of an antibacterial agent that prevents the appearance of visible growth of a microorganism. Moreover, the same procedure to determine the minimum bactericidal concentrations (MBCs) was used. The MIC and MBC values were determined against two bacterial strains, *Staphylococcus aureus* (ATTC 6338) and *Staphylococcus epidermidis* (ATCC 12228). Prior to the experiments, bacterial strains suspended in Mueller–Hinton broth were adjusted to a concentration of 1∙10^6^ CFU/mL. In general, a two-fold dilution series from silver nanoparticles suspensions in Mueller–Hinton broth were prepared to give working concentrations ranging between 256–0.125 mg/mL. Subsequently, 50 μL of each AgNPs dilutions were dispersed into microdilution trays. An equal volume of inoculum was added to each well of the tray, resulting in a final concentration of 5·10^5^ CFU/mL of bacterial strains. The prepared plate was incubated for 18 h at 37 °C in aerobic conditions. All determinations were performed in triplicate.

### 2.9. Statistical Analysis

Biological studies were performed in four replicates each and at least two biological repeats. Inter-group outcomes were analysed by a one-way ANOVA (analysis of variance) with post hoc Dunnett’s test. Intra-group differences were assessed using the non-parametric Mann–Whitney U test. In all cases, the significance was accepted at *p* < 0.05. Statistical analysis was performed using GraphPad Prism v.7 (GraphPad Software, San Diego, CA, USA).

## 3. Results

### 3.1. Extract and Infusion Antioxidant Properties

The polyphenolic compounds contained in plants are well known for their oxygen-scavenging properties and reducing effect. Therefore, it was necessary to ascertain the total amount thereof in the prepared extract and infusion. The TPC (expressed as milligrams per gram of the dry weight) and scavenging-effect results of the *C. incanus* extract and infusion are summarized in Table 1. The TPC, as well as antioxidant activity results, showed differences between extract and infusion antioxidant properties. As can be seen, the *C. incanus* infusion was a more effective DPPH radical scavenger, compared with its extract. The obtained data also showed that the TPC of plant infusion was significantly higher than those obtained for plant extract.

### 3.2. Characterization of Silver Nanoparticles

Forming of AgNPs with the use of various volumes of plant extracts and infusions applied as capping and reducing agents, and NP suspensions’ time-stability, were monitored by UV–visible spectrophotometer, at a wavelength range from 300 nm to 750 nm. Surface plasmon resonance (SPR) of AgNPs due to the oscillation of free electrons (plasmons) in the NPs was recorded as an intense absorption band in the range of wavelengths between 380 nm to 450 nm.

**Figure 1:**
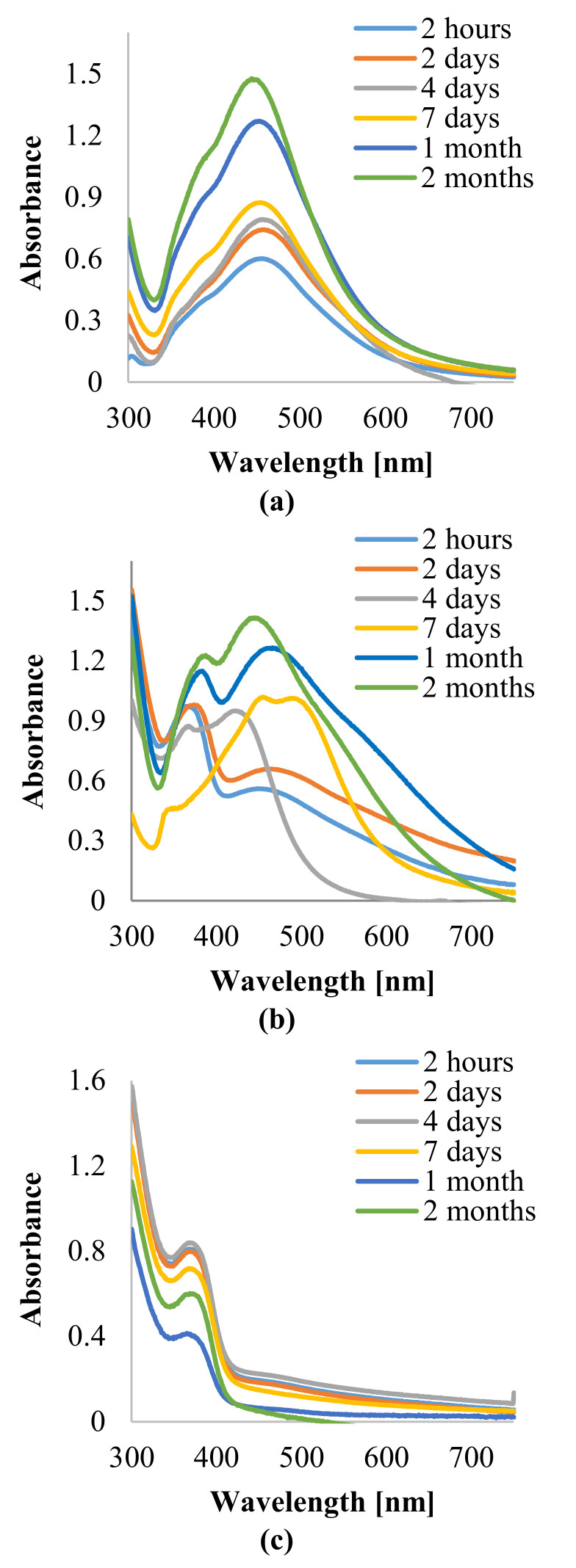
Absorption spectra of AgNPs prepared with the use of a *C. incanus* leaves extract (**a**) 5 mL, (**b**), 10 mL, (**c**) 15 mL recorded at various time intervals.

**Figure 2:**
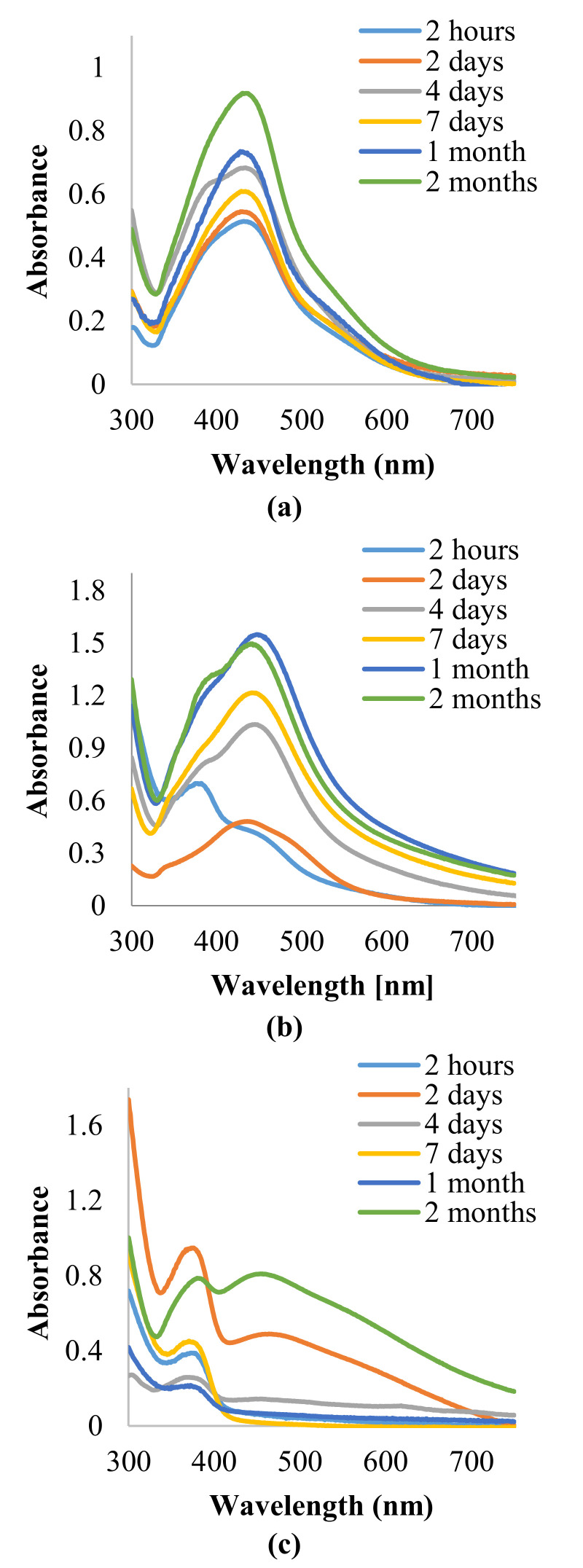
Absorption spectra of AgNPs prepared with the use of a *C. incanus* leaves infusion (**a**) 5 mL, (**b**), 10 mL, (**c**) 15 mL recorded at various time intervals.

UV–Visible absorption spectra of AgNPs synthesized by the reduction of AgNO_3_ with 5 mL, 10 mL, and 15 mL of the obtained plant extract and infusion, at a constant concentration of AgNO_3_, are presented in Figure 1 and Figure 2, respectively.

The recorded absorption spectra revealed that the AgNPs formation with the use of the lesser amount of aqueous extract and infusion was more efficient as compared with syntheses performed with the use of 10 mL and 15 mL of reducing agent. Moreover, it is shown that, as the extract and infusion content increases, the maxima of the SPR absorption bands of NP_S_ were placed at longer wavelengths, except for sample 1c, for which the maximum of absorption band was centered at 369 nm. However, the absorption bands of specimens 1c and 2c were less intense as compared with other AgNPs suspensions, which suggests that the formation of nanoparticles in these cases was less efficient. Additionally, absorption bands of samples prepared with 10 mL of extract and infusion were nonsymmetric and wider in comparison with AgNPs suspensions obtained with 5 mL of phytochemical solution, which points out the synthesized nanoparticles were polydisperse. During further observation, significant shifts in bands’ maxima were not observed.

**Figure 3:**
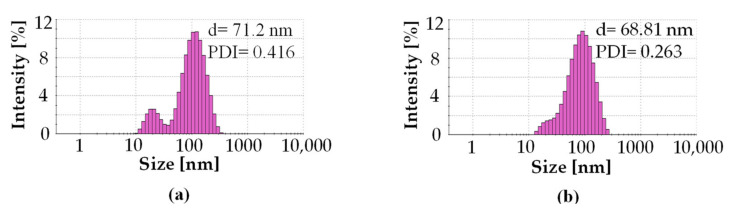
DLS histograms of AgNPs prepared with 5 mL of extract (**a**) and 5 mL of infusion (**b**) of *C. incanus*.

AgNPs’ average size distribution, as well as their polydispersity index (PDI) in the samples prepared with 5 mL of extract and 5 mL of infusion, were determined by the dynamic light scattering (DLS) method. The particles intensity-averaged intensity distributions histograms, polydispersity indexes, and the average diameters of the AgNPS are presented in Figure 3. The mean average nanoparticle diameter (d) was 71.2 nm for AgNPs prepared with extract, and 68.8 nm for the second sample. AgNPs suspensions obtained with the use of 5 mL of extract (3a) showed a bimodal distribution. The main peak mode was placed at ~110 nm, and a second peak mode at 20 nm. Particle size distribution was in the range of 10 nm to 350 nm. AgNPs suspensions prepared with infusion (3b) revealed monomodal distribution, and a narrower particle-size distribution, ranging from 15 nm to 180 nm, with the maximum intensity peak mode centered at 90 nm. Moreover, PDI of sample 3b (0.3), was relatively low as compared with 2a sample PDI (0.4).

**Figure 4:**
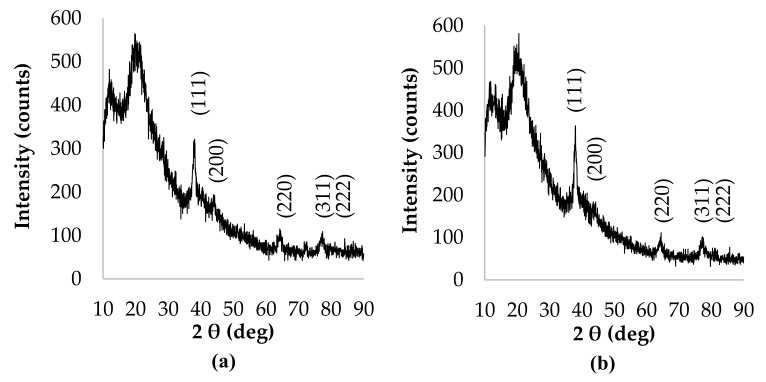
XRD diffraction patterns of AgNPs prepared with 5 mL of extract (**a**) and 5 mL of infusion (**b**) of *C. incanus*.

Figure 4 presents the X-ray diffraction (XRD) patterns of AgNPs obtained with *C. incanus* extract (4a) and infusion (4b). Bragg reflections referring to the (111), (200), (220), (311), (222) sets of lattice planes were registered. Based on the XRD patterns the face-centred cubic (fcc) cell of Ag with the lattice constant a = 4.0862 Å were indicated. XRD analysis confirmed crystalline nature of the prepared AgNPs. However, the intensities of the peaks assigned to AgNPs were greater for those prepared with the infusion, indicating a greater degree of crystallinity.

**Figure 5:**
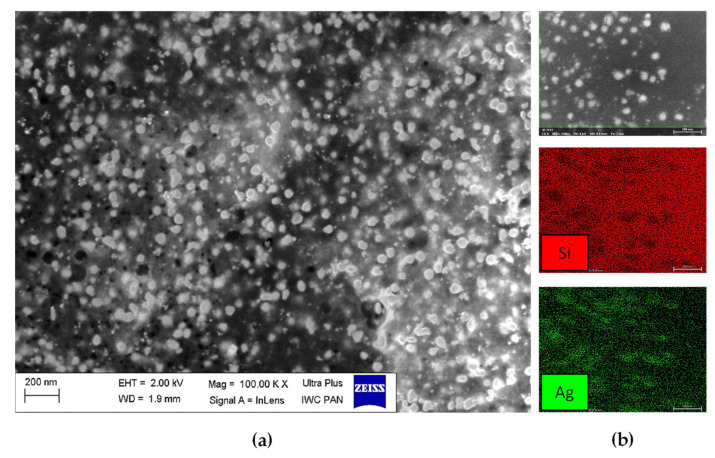
SEM image (**a**) and EDS mapping of the elemental species (**b**) of AgNPs sample prepared with 5 mL of infusion of *C. incanus*.

Scanning electron microscope was employed to visualize size and shape of the prepared AgNPs. The morphologies of the AgNPs obtained with *C. incanus* aqueous infusion (5 mL) are presented in Figure 5a, and EDS mapping of the elemental species on the specimen surface is shown in Figure 5b. SEM-EDS analysis confirmed formation of silver nanoparticles with the sphere-like morphology. Additionally, differences in NPs’ size were noted, which is consistent with the DLS measurement.

### 3.3. FT-IR Analysis

**Figure 6:**
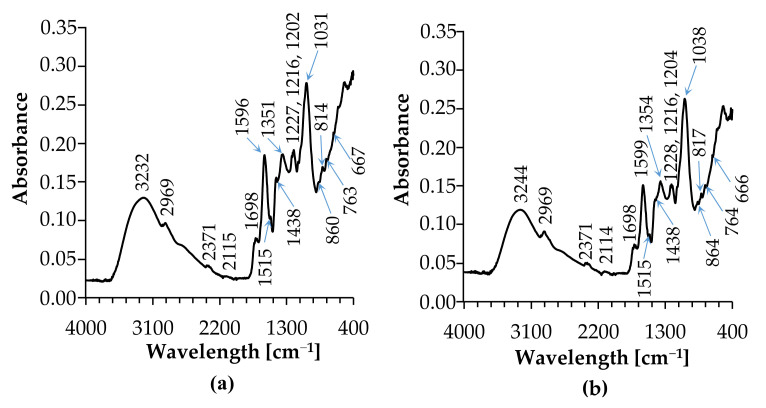
FTIR spectra of *C. incanus* leaves extract (**a**), and infusion (**b**).

The FT-IR spectral analysis was utilized to identify functional groups of the active components present in the extract and infusion based on the location of the peaks in the region of IR radiation. Figure 6. shows the FT-IR spectra of *C. incanus* leaves extract and infusion. The results of FT-IR analysis confirmed the presence of OH, N-H, -CH_3_, C=C and C-C of the aromatic ring, C-O, C-O-H (deformation of phenols), C-O-C (cyclic ethers, phenols), C-N stretching (aliphatic primary amine), C-O (Arom-O), C-Cl, and N-C=O (amides) functional groups.

FTIR peaks are assigned for stretching and bending vibrations that indicate particular functional groups. The FTIR absorption bands of the *C. incanus* leaves extract and infusion are assigned and summarized in Table 2.

The 3232–2114 cm^−1^ range corresponds to stretching of OH and NH groups, and C-H stretching of -CH_3_ and-CH_2_ groups. The ranges 1599–1515 cm^−1^ and 1438–1435 cm^−1^ are characteristic of the stretching of C=C and C-C conjugated with C=C of the aromatic ring. Bands located at 1438–1435 cm^−1^ correspond to deformations of C-H and CH_2_-OH groups. Another characteristic band assigned to deformation of C-O-H of phenols is placed at ~1227 cm^−1^. The 1216–1202 cm^−1^ bands are assigned to C-H, CH_3_, and aromatic C-H deformations, whereas bands placed at 763–666 cm^−1^ range correspond to stretching vibration of C-O bonds belonging to ester, ether, or phenols, C-O-C of the large rings, out-of-plane bending of aromatic C-H, and bending vibration of the CH-tri-aromatic substitution group. Similar FT-IR spectra were recorded by Latos-Brozio for *Cistus Linnaeus* raw plant material [47].

### 3.4. Direct Contact Cytotoxicity Assay

**Figure 7:**
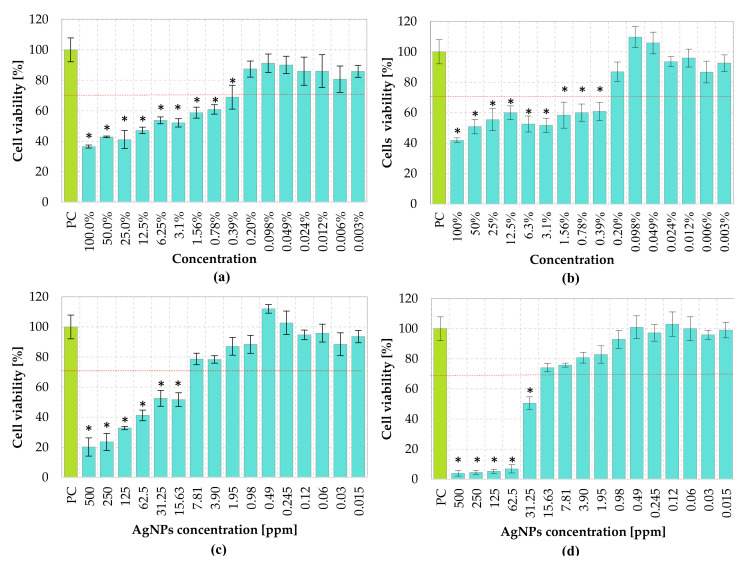
L929 fibroblasts viability (MTT assay) treated with the *C. incanus* extract (**a**), infusion (**b**), AgNPs prepared with extract (**c**), and AgNPs prepared with infusion (**d**). The values show cell viability calculated in comparison to cells cultured in medium (100% viability). Statistically significant (*p* < 0.05) differences are marked with *.

In this research, in order to determine the viability of cells exposed to *C. incanus* infusion, extract and AgNPs obtained with 5 mL of extract and 5 mL of infusion, a dilution series in the range of 0.003% to 100% or 0.015 ppm to 500 ppm, respectively, were prepared (Figure 7).

As stated in the ISO standard, a tested component is recognized as cytotoxic when cell viability is reduced by more than 30%. The obtained results showed that the *C. incanus* extract and infusion, at a concentration range from 0.39% to 100%, caused a significant cytotoxic effect, leading to reduced viability of the fibroblasts in comparison with cells cultured in the medium (*p* < 0.05). However, the cytotoxic effect was not observed for the *C. incanus* extract and infusion used in concentrations equal to or lower than 0.2%. As stated in ISO standard, a component with biomedical applications is non-cytotoxic when, after 24 h incubation, cells viability remains higher than 70%. Considering this criterion, as well as statistical analysis, *C. incanus* extracts and infusions at concentrations below 0.39% should be recognized as safe at the in vitro cellular level. Similarly, cytotoxicity assessment of AgNPs suspensions revealed a dose-dependent cytotoxic effect, although with lower intensity than the *C. incanus* extract and infusion. AgNPs, in a concentration range of 15.63 ppm to 500 ppm and prepared with the use of a plant extract, induced a significant reduction in cell viability when compared with control cell cultures (*p* < 0.05). Plant fusion-obtained AgNPs, below concentrations of 15.6 ppm, were showed to be non-cytotoxic towards reference cell line.

### 3.5. Pro-Inflammatory Assay

**Figure 8:**
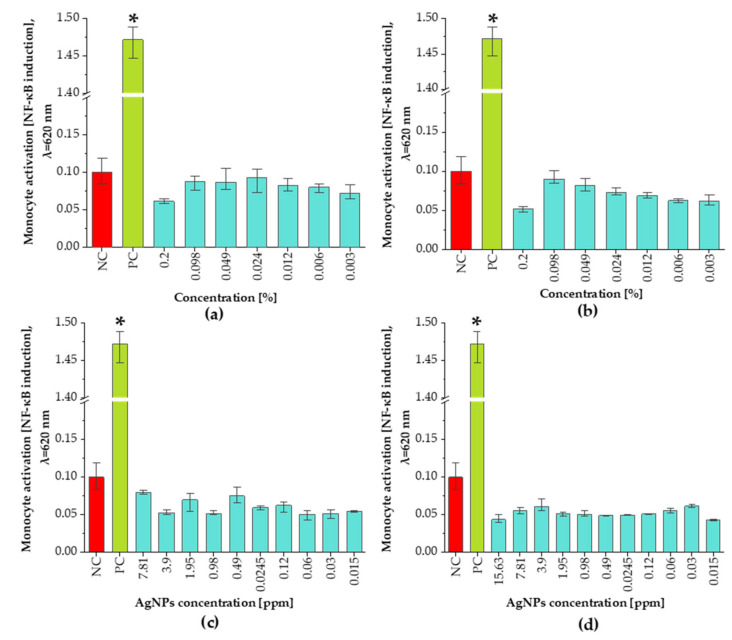
Immunocompatibility test results of *C. incanus* extract (**a**), infusion (**b**), AgNPs prepared with extract (**c**), and AgNPs obtained with infusion (**d**) toward human monocytic THP1XBlueTM cells compared with the positive control (PC) and untreated cells (NC). Statistically significant differences (*p* < 0.05) calculated in comparison to untreated cells (NC) are marked with *.

As shown in Figure 8, the results obtained in the immunostimulatory assay demonstrated that neither *C. incanus* extract/infusion, nor plant-derived NPs exhibited pro-inflammatory potential towards THP1-XBlue^TM^ monocytes. In comparison, the monocytes treated with standard endotoxin from Gram-negative bacterium—LPS of *Escherichia coli*, significantly (*p* = 0.004) induced the activation of THP1-XBlue^TM^ monocytes, when compared to non-treated cell cultures. Similarly, the immunocompatibility assay showed that none of the plant extracts generated an activation of a human monocyte cell line in all concentration ranges, and all the obtained values remained approximately on the level of untreated cultures.

### 3.6. Antimicrobial Activity of AgNPs

The in vitro evaluation of antimicrobial property of AgNPs was performed according to the microdilution method. The experiment showed that NPs prepared from extract and infusion are promising agents for inhibiting the growth of both tested bacterial strains (Table 3). Notwithstanding this, the MIC of NPs obtained with infusions was lower (2 ppm) for *S. aureus* in comparison with MIC for *S. epidermidis* (16 ppm). Furthermore, it was proved that antimicrobial activity expressed as MBC for NPs prepared with infusion was 128 ppm for both strains tested. It is also worth emphasizing that all determined MIC and MBC values were greater for NPs obtained with plant extract.

## 4. Discussion

In this study, the F-C procedure and DPPH free radical reduction assays were applied to characterize the antioxidant properties of an aqueous extract and infusion of *C. incanus*. The analysis of TPC, as well as antioxidant activity of extract and infusion, revealed compelling differences in the measured values dependent on the applied extraction method. The analysis revealed that higher phenolic compounds content (85.97 ± 6.54 mg GAE·g^−1^ d.w.), as well as stronger antioxidant activity (10.76 ± 0.59 mg/mL), were observed for the plant’s infusion. A lower value of TPC (12.65 ± 1.04 mg GAE·g^−1^ d.w.), together with RSA (3.10 ± 0.14 mg/mL) of *C. incanus* extract, may be associated with the decomposition of the thermolabile compounds caused by the exposition of plant leaves to an extracting medium at a boiling point for a longer time, in comparison with the infusion preparation method. The effect of the extraction methods on TPC and antioxidant activity has been studied extensively by many researchers. The presented results clearly indicate that there is strong dependence between various extraction factors (extraction time, temperature, type of solvent and its pH, sample quantity) and TPC and antioxidant capacity of plant extracts [48,49,50,51]. Petkova et al. compared the antioxidant activity and the TPC in infusions and microwave-assisted extracts from herbs showing that utilization of second extraction method leads to greater values of both extract parameters [50]. The influence of heating temperature and time of heating on TPC on antioxidant capacity was examined by Ross et al., indicating significant decreases in these parameters’ values with increasing temperature and time of processing [52]. On the other hand, a comparative study on TPC and total antioxidant content of the *Cistus creticus* when hot-and-cold brewed showed that the total radical-scavenging activities of brews were similar, however the TPC was greater for hot brewing [53].

*C. incanus* leaf extract and infusion were proposed as potential reducing and stabilizing mediums in AgNPs obtaining taking into consideration its availability and health-promoting properties. The performed experiments confirmed the formation of nanometric Ag structures. Spectrophotometric investigation revealed a distinctive absorption band in the wavelength range of 380 nm to 450 nm, as a result of surface plasmon excitation. Additionally, this analysis confirmed that both infusion and extract were able to reduce Ag^+^ to Ag^0^ and also stabilize the obtained nanoparticles. Thus, it can be deduced that extraction of *C. incanus* with water causes an isolation of antioxidants with stabilizing capability. Phytochemicals extracted from the plant act as reducing and stabilizing agents in the process of reduction of silver ions from silver nitrate solution into silver nanoparticles; however, the exact mechanism of the conversion remains unclear. An analysis of nanoparticles size distribution was performed with the DLS method, which allowed the measuring of the diameters of particles, up to 1–2 nm. Nevertheless, it is worth noting that the DLS method provides information on the particle hydrodynamic diameter encompassing the particle itself, as well as stabilizing agents residing on its surface. Moreover, it has been proven that the particles-size measurement results are dependent on the applied research method [54]. The conducted investigation clearly indicates that the NPs size measured with the DLS method is slightly larger in comparison with the sizes determined by the TEM and XRD techniques. However, due to its facility and availability, this technique remains mostly used in nanoparticles size measurement. The DLS measurement revealed that the particles size distribution of AgNPs prepared with plant extract was of bimodal distribution, whereas AgNPs obtained with *C. incanus* infusion revealed monomodal distribution. Furthermore, the values of intensity-average diameter and PDI were lower for the sample prepared with the infusion (d = 68.8 nm; PDI = 0.3) than NPs synthesized with extract (d = 72.1 nm; PDI = 0.4). Additionally, it is shown that the size-distribution maximum intensity for AgNPs suspensions obtained using a *C. incanus* infusion was located at a lower value of diameter when contrasted against NP suspensions prepared with the extract. This can be explained by less effective nanostructures stabilizing effect caused by lower TPC content in the extract as compared with infusion. The X-ray diffraction analysis of the NPs samples showed that the nanostructures characterized with face-centered cubic (fcc) crystal system were formed. The diameter of AgNPs determined with SEM was found to be lower than 100 nm. The obtained nanostructures were close to being spherical in shape.

For biocompatibility assessments of the infusion, extract, and NPs, direct contact cytotoxicity assay was used. The conducted biocompatibility assessment disclosed that cytotoxicity of AgNPs and phytochemical solutions are dose-dependent, and AgNPs prepared with 5 mL of infusion induced the cytotoxicity as high as 96.1 ± 1.8%. This notwithstanding, AgNPs of concentrations lower than 7.81 ppm obtained with *C. incanus* extract and ~16 ppm for NPs prepared with infusion did not cause a cytotoxic effect in the treated fibroblast cell line. Składanowski et al. showed that AgNPs at concentration 1 and 5 µg/mL do not cause any cytotoxic effect on L929 murine fibroblast [55]. Conversely, a research performed by Veeraraghavan et al. revealed that AgNPs do not induce cytotoxicity against the fibroblast cells at a concentration of 15 µg/mL [56]. Discrepancies in the results presented in different scientific reports may be the result of size-dependent cytotoxicity of NPs, as reported by Carlson et al. and Zhao et al. [57,58].

For *C. incanus* infusion and extract an acceptable value of viability of the treated cells (70% in accordance with the ISO standard) was reached only for solutions diluted to 0.2%. The cytotoxic effect of the phytochemicals solutions used at higher concentrations may be caused by the strong activity of organic compounds, resulting in cell lysis or metabolic activity inhibition. It is worth highlighting that plant solutions at a concentration range of 0.39% to 1.56% induce cytotoxic effects, whereas corresponding AgNPs concentrations remain nontoxic. This effect may be a result of the oxidation of some plant-derived compounds during the reduction of Ag ions to AgNPs, leading to the formation of less or nontoxic products. Numerous studies indicate that AgNPs toxicity is associated with the interaction of nanoparticles with cell membranes, resulting in an altering of their permeability, leading to cell death [59,60]. In accordance with research performed by the Authors recently, it is shown that the prepared AgNPs were nontoxic at higher concentrations when compared to Ag ions from AgNO_3_ used as a silver source in nanoparticle preparation in this experiment [31]. The cytotoxicity of silver ions has been thoroughly tested in many experiments, proving their dose-dependent toxicity [61]. Liu et al. [62] proved that Ag^+^ at a concentration of 1 ppm causes death of 80% of tested L929 cells, whereas Ag^+^ at a higher concentration (10 ppm) results in a whole treated cell colony death. In our studies, the cytotoxic effect of NPs prepared with *C. incanus* extract and infusion was compared. The viability of L929 fibroblasts treated with 500–15.6 ppm NPs prepared with extract showed a low percentage of viable cells (20.15 ± 6.0%–51.64% ± 4.6%). Also, the AgNPs obtained with infusion in concentrations of 500–31.25 ppm induced a noticeable reduction in cells viability (3.9 ± 1.8.5%–50.5 ± 4.2%), in comparison with the positive control (untreated cells). Viability exceeded 70% was recorded in cultures incubated with AgNPs at concentrations ≤7.8 ppm (82.1 ± 1.1%) synthesized with extract, whereas there was no cytotoxic effect observed for AgNPs obtained with an infusion at concentrations ≤15.6 ppm.

We report that an application of the obtained NPs, as well as plant extracts and infusions, do not result in the activation of monocytes. Anti-inflammatory properties of *C. incanus* extract are well-reported and can be attributed to the activity of some organic compounds such as gallic acid, rutin, and myricitrin, which were found to be components of this plant extract [63,64]. However, some studies showed that AgNPs used at a concentration ranging from 10 μg/mL to 100 μg/mL caused induction of NF-κB transcription factor [55]. Discrepancies between the results of the experiments may be due to various reducing agents applied, as well as microbial contaminants which may be transferred during nanoparticles generation. The results obtained by Zhu et al. [65] showed that gallic acid, a component of *C. incanus* extract, demonstrates anti-inflammatory activity via inhibiting NF-κB pathway in ulcerative colitis. Thus, it can be concluded that the proposed plant extracts may inhibit potential AgNPs-mediated activation of monocytes. To determine the activity of the NPs on certain bacterial strains, a microdilution test was used and the minimum inhibitory concentration (MIC) and the minimum bactericidal concentration (MBC) were determined. The result shows that NPs prepared with *C. incanus* infusion at concentrations of 2 ppm and 16 ppm can be applied as potential agents for inhibiting the growth of *S. aureus* and *S. epidermidis*, respectively. However, the MIC values for NPs obtained with plant extract were greater as compared with NPs utilized with the infusion. Consequently, to achieve an appropriate NP antibacterial efficiency, a greater concentration of NPs is required. Although ppm application of NPs prepared with *C. incanus* extract, considering their cytotoxic effect at concentrations greater than 7.81 ppm application of NPs prepared with *C. incanus* extract, may prove to be impossible, on the other hand, the MIC values of NPs obtained with infusion guarantee their safety. For both tested samples, the concentrations defined as MBC are not acceptable, given the results of the applied cytotoxicity tests. Evaluation of antibacterial activity of AgNPs synthesized from *Streptomyces* sp. NH28 strain against *Staphylococcus aureus* (ATTC 6338) strain was performed by Składanowski et al. [55] and the MIC of the tested NPs was in the range of 1.25–10 μg/mL. In contrast, research performed by Sharifi-Rad et al. showed that MIC for *S. aureus* (ATCC 25923) strain was 37.5 μg/mL for AgNPs prepared with *Otostegia persica* leaf extract [66]. Such differences may be related to the different size of nanoparticles, as many studies have indicated [67,68].

## 5. Future Perspectives

The presented method of AgNPs obtaining can be utilized in the preparation of a safe medical agent which is worthy of further examination as a potential agent inhibiting various bacteria and tumour cells growth. It is also worth considering the use of nanoparticles as an active substance in drug carriers, components of wound dressings intended for the treatment of difficult-to-heal wounds, or to modify the surface of implants preventing bacterial infections. The proposed method of AgNPs preparation is fast and does not require the usage of any additional stabilizing agents or hazardous solvents. These features make the described method both environmentally friendly and economically viable.

## 6. Conclusions

This paper describes inexpensive, environmental-friendly, and facile method of AgNPs preparation using *Cistus incanus* leaves aqueous extract and infusion at various concentrations. The presented method of AgNPs preparation stays in line with current green chemistry trends. Phytochemicals contained in extract and infusion acted as reducing and capping agents deeming application of additional chemicals unnecessary. The obtained NPs were spherical in shape with the average size in the 68.8–72.1 nm range. The biocompatibility test showed that AgNPs at concentrations lower than 7.81 ppm obtained with *C. incanus* extract and 16 ppm for NPs prepared with infusion did not induce cytotoxicity. Moreover, the tested AgNPs have an antibacterial effect against *S. aureus* and *S. epidemirdis* strains and the minimum inhibitory concentration for NPs obtained with plant infusion was lower, as compared with NPs synthesized with the extract. In conclusion, we hope that the presented results will help to develop a broader spectrum of potential applications of AgNPs with the proposed method.

## Figures and Tables

**Table 1:** TPC and antioxidant capacity of a *C. incanus* extract and infusion.

Title 1	Phenolic Content (mg GAE·g^−1^ d.w.) ± SD	Antioxidant Activity IC_50_ (mg/mL) ± SD
*C. incanus* extract	12.65 ± 1.04	3.10 ± 0.14
*C. incanus* infusion	85.97 ± 6.54	10.76 ± 0.59

**Table 2:** IR band assignment of the *C. incanus* leaves extract and infusion.

Infusion [cm^−1^]	Extract [cm^−1^]	Peak Assignment
3232	3244	OH stretching, N-H stretching [30]
2969	2969	C-H symmetric stretching of -CH_3_, OH stretching [30]
2930	2925	C-H stretching of vibration of methyl and methoxy groups, stretching vibration of -CH_3_ or -CH_2_ groups (carboxylic acid), OH stretching [32]
2371	2371	-CH_3_ and -CH_2_ stretching [33]
2115	2114	-CH_3_ and -CH_2_ stretching [33]
1698	1698	C=O stretching (ketone) [34]
1596	1599	stretching C=C (aromatic ring) [35]
1515	1515	C=C (aromatic ring) [36]
1438	1435	C-C aromatic (conjugated with C=C), O-H bending [37]
1351	1354	C-H deformations, CH_2_-OH deformation, N-H stretching [38]
1227	1228	C-O (alcohol hydroxyl group), C-O-H (deformation of phenols) [39]
1216	1216	C-H deformations [39]
1202	1204	NH_2_ in-plane bending, aromatic C-H [40]
1141	1140	in—plane bending, CH_3_ deformation [41]
1031	1038	N-H symmetric bending, O-H bending [42]
860	864	C-O stretching benzene nucleus, C-O stretching of C-OH [43]
814	817	C-O-C (cyclic ethers, large rings) [43,44]
763	764	stretching vibration of C-O bonds, C-O-C (ester, ether, or phenols), C-N stretching (aliphatic primary amine) [44,45]
667	666	aromatic C-H out-of-plane bending, bending vibration of the CH‒tri-aromatic substitution group [46]

**Table 3:** The antimicrobial activity expressed as minimum inhibitory concentration (MIC) and minimum bactericidal concentration (MBC), respectively.

Sample	*S. aureus*		*S. epidermidis*	
	MIC [µg/mL]	MBC [µg/mL]	MIC [µg/mL]	MBC [µg/mL]
Extract NPs	>256	>256	32 ppm	128 ppm
Infusion NPs	2 ppm	128 ppm	16 ppm	128 ppm

## Data Availability

The data that support the findings of this study are contained within the article.
